# Long-term, interventional, open-label extension study evaluating the safety of tocilizumab treatment in patients with polyarticular-course juvenile idiopathic arthritis from Poland and Russia who completed the global, international CHERISH trial

**DOI:** 10.1007/s10067-018-4071-9

**Published:** 2018-04-13

**Authors:** Violetta Opoka-Winiarska, Zbigniew Żuber, Ekaterina Alexeeva, Vyacheslav Chasnyk, Irina Nikishina, Grażyna Dębowska, Elżbieta Smolewska

**Affiliations:** 1 Department of Pediatric Pulmonology and Rheumatology, Medical University of Lublin, Gębali 6 Street, 20-093 Lublin, Poland; 2 Department of Pediatric Neurology and Rheumatology, St. Louis Children’s Hospital, Cracow, Poland; 3 Federal State Autonomous Institution “National Medical Research Center of Children’s Health” of the Ministry of Health of the Russian Federation, Federal State Autonomous Educational Institution of Higher Education I.M. Sechenov First Moscow State Medical University of the Ministry of Health of the Russian Federation, Moscow, Russia; 4 St. Petersburg State Pediatric Medical Academy, St. Petersburg, Russia; 5 Pediatric Department, Federal State Budgetary Institution V.A. Nasonova Research Institute of Rheumatology, Moscow, Russia; 6 Medical Department, Roche Poland Sp. z o.o. (the employee of Roche Polska Sp. z o.o. till 30.04.2015), Warsaw, Poland; 7 Department of Pediatric Rheumatology, Medical University of Lodz, Lodz, Poland

**Keywords:** Biologicals, Juvenile idiopathic arthritis, Tocilizumab, Treatment

## Abstract

Efficacy and safety of tocilizumab (TCZ), an interleukin-6 receptor inhibitor, were demonstrated in juvenile idiopathic arthritis (JIA) with polyarticular course (pJIA) in the CHERISH trial. This observational, III phase study evaluated long-term treatment of TCZ in pJIA patients was conducted by members of the Pediatric Rheumatology International Trials Organization (PRINTO) from Poland and Russia. Forty-one patients, who had completed the CHERISH core study (104 weeks), were extensionally treated with TCZ (8 mg/kg, intravenous infusion every 4 weeks). Total treatment time was from 131 to 193 weeks. The long-term safety (the primary endpoint) and efficacy were evaluated. All patients achieved ACR70 response in the core study and continued to achieve at least ACR50 response up to week 24 of this study. The safety population comprised 46.41 patient-years (PY). Rates per 100 PY of adverse (AEs) and serious events (SAEs) were 181.0 and 6.46, respectively. Pharyngitis and respiratory tract infections were the most common AEs. Except one AE (severe neutropenia), all others were classified as mild (24.4%) or moderate (29.3%). The incidence of SAEs was low (7.3%). No new safety findings were observed. The safety profile of over 2.5-year treatment with TCZ is consistent with the pre-marketing CHERISH clinical trial. Presented data and continued efficacy response support the use of TCZ in pJIA. EUDRACT No: 2011-001607-12. https://clinicaltrials.gov/ct2/show/study/NCT01575769?term=ML27783

## Introduction

Juvenile idiopathic arthritis (JIA) is a clinically heterogeneous group of diseases characterized by arthritis that begins before 16 years of patient age and persists for minimum 6 weeks. The majority of children with JIA have a polyarticular form of the disease (pJIA) defined as arthritis in more than four joints during their disease course [1, 2]. The course of pJIA is characterized by frequent relapses, long periods of active disease, higher risk of joint damage, low treatment effects, and reduced quality of life [2, 3]. A variety of therapies are currently used in the treatment of pJIA, including both non-biologic and biologic disease modifying anti-rheumatic drugs (DMARDs) [4].

Tocilizumab (TCZ), a humanized, monoclonal anti-IL-6 receptor (IL-6R) antibody binding to membrane and soluble IL-6R, inhibits IL-6-mediated signaling [4]. TCZ showed safety and activity in several studies in patients with systemic and polyarticular course of JIA [5–9]. Efficacy and safety of TCZ were demonstrated in the global, three-part, randomized, placebo-controlled, double-blind, withdrawal, phase III clinical trial (the CHERISH study) in patients with active pJIA (rheumatoid factor-positive or rheumatoid factor-negative pJIA or persisting oligoarticular JIA) for at least 6 months and inadequate response to methotrexate (MTX) [5]. The first part of the CHERISH study was a 16-week open-label phase in which 188 patients received intravenous TCZ every 4 weeks. The improvement was evaluated by JIA ACR (the American College of Rheumatology) response. One hundred sixty-three patients, who achieved at least JIA ACR 30 responses, were enrolled into the second part of study. It was 24-week double-blind phase with randomization 1:1 to placebo or to TCZ. The primary endpoint was JIA flare, compared with week 16. Patients flaring or completing part 2 received open-label TCZ up to week 104 of study. Published results of the CHERISH study demonstrated efficacy of TCZ therapy up to 40 weeks (parts 1 and 2) of treatment [5]. European Medicines Agency has approved TCZ to treat children with pJIA in 2013.

The purpose of the current analysis was to evaluate the long-term safety and efficacy of extensively prolonged TCZ therapy in 41 patients with pJIA from Poland and Russia who had completed the CHERISH core study. Total TCZ treatment time (with the core study) was from 131 to 193 weeks. Up to now, this is the longest published observation of patients with pJIA treated with TCZ.

## Methods

### Patients and study design

This long-term, interventional, open-label extension study evaluated the safety of TCZ treatment in patients with pJIA from Poland and Russia who completed the CHERISH study. This study, as the core trial, was conducted by members of PRINTO from Poland and Russia. This was accordant with the Declaration of Helsinki, good clinical practice guidelines, local requirements, and approved by central IRB/IEC (Komisja Bioetyczna przy Uniwersytecie Medycznym w Lublinie, Poland).

The main inclusion criterion was the completion core, CHERISH study with at least JIA ACR 30 clinical response to TCZ with no adverse events (AEs), serious adverse events (SAEs), or conditions that could lead to unacceptable risk of continued treatment.

A total of 46 patients with pJlA from Poland and Russia who participated in the core CHERISH study were planned to be enrolled into this trial. Two patients from the CHERISH study refused further participation. Forty-three patients and parents agreed to continue the study and proceeded to screening. Two patients failed screening: one patient due to low neutrophil count and white blood cell count and one patient due to JIA ACR below 30.

Written informed consent for the extension phase study participation was obtained from patients at the age 18 or older. For younger patients, written consent was signed by the one of the parents or legal guardian.

Finally, 41 patients were enrolled, 18 from Poland, and 23 from Russia. All those patients completed 104 weeks of treatment in the CHERISH study with at least JIA ACR 70 response to TCZ. Patients experienced no AEs, SAEs, or other conditions that could lead to the unacceptable risk of treatment continuation. Thus, all patients were eligible to continue the treatment (TCZ 8 mg/kg in intravenous infusion every 4 weeks) within this long-term extension study. Initially, the trial was planned for 104 weeks or until TCZ was commercially available for pJIA, whichever occurred first. The first TCZ dose in this regional extension protocol was to be administered at week 104 of the core study—at the last visit of the core study. All patients could have received ongoing MTX, non-steroidal anti-inflammatory drugs (NSAIDs), prednisone, or corticosteroid equivalents at the stable pre-entry doses during the study as the background medication. Patients treated with MTX also received either folic acid or folinic acid according to the local standard of care.

The extension study was terminated both in Russia and in Poland due to the fact that TCZ was approved in both countries for the new indication, i.e., pJIA. That was in accordance with the timeline provided in the study protocol and informed consent forms. As the result of the early termination, the follow-up period in extension study varied from 27 to 89 weeks. Total TCZ treatment time was therefore from 131 to 193 weeks (with the core study).

The long-term safety of TCZ treatment (the primary endpoint) was evaluated for the entire study process (27 to 89 weeks depend on the patient) and follow-up visit. For the purpose of efficacy endpoint analysis, data for the first 24 weeks in which all patients remained in the study are presented in this report. Efficacy was assessed in relation to the first visit in the core study. There, adding together, our study assesses the effectiveness of the entire TCZ treatment process lasting a total of 128 weeks. Later time points are referenced only. The publication the CHERISH study presented efficacy results of the first 40 weeks and safety results of entire study [5].

### Assessment and outcomes

Clinical assessments (baseline and every 4 weeks thereafter) included six JIA core response variables: number of joints with active arthritis, number of joints with limitation of motion (LOM), physician global assessment (PGA) of disease activity (range, 0–100), assessment of patient overall well-being (range, 0–100), physical function measured by the Childhood Health Assessment Questionnaire-Disability Index (CHAQ-DI; range, 0–3), and erythrocyte sedimentation rate (ESR) [10]. Clinically inactive disease was defined as PGA indicating no disease activity and the absence of all of the following: joints with active arthritis, uveitis, and ESR greater than 20 mm/h [11].

### The primary endpoints

The primary endpoint was to evaluate the long-term safety of TCZ treatment in patients with pJIA who entered this extension study. The primary safety outcome measures included the number and percentage of AEs, SAEs, AEs of special interest, and study drug-related AEs. Other safety endpoints included clinical laboratory evaluations, physical examination, vital signs, height/weight, electrocardiography (ECG), tuberculosis (TB) test, chest X-ray, slit-lamp ophthalmology examination, and recording of hospitalizations/deaths. A tuberculin test, either protein derivative tuberculin skin test (PPD) or interferon-based test and chest X-ray, was performed yearly during the whole period of TCZ treatment, due to safety reasons. Pneumococcal vaccination has not been performed in the study group.

### Secondary endpoints

The secondary endpoint was to investigate the long-term efficacy during treatment with TCZ in the study group.

The efficacy was assessed in relation to the first visit of the core study. The following parameters were evaluated at visits: baseline (during the last visit of core study) and every 4 weeks thereafter to 24 week of study:

- The proportion of patients with JIA ACR responses. The JIA ACR30/50/70/90 response was defined as three of any six core outcome variables improved by at least 30%/50%/70%/90% from the baseline assessments, with no more than one of the remaining variables worsened by more than 30%. Patients were required to have at least JIA ACR 30 clinical response to TCZ at week 104 of the core study to continue to this extension study.
- The proportion of patients with inactive disease and clinical remission (CR) at visits.

Four levels of CR were defined for patients during TCZ treatment in this study:

1. CR on medication for a minimum of 6 consecutive months, while the patient was on medication
2. CR off corticosteroid medication (still on TCZ) for a minimum of 6 consecutive months, while the patient was off corticosteroid medications
3. CR off both corticosteroid and MTX medication (still on TCZ) for a minimum of 6 consecutive months, while the patient was off both MTX and corticosteroids
4. CR off all anti-inflammatory medications (still on TCZ) for a minimum of 6 consecutive months, while the patient was off all anti-arthritis medications (corticosteroids, MTX, NSAIDs).

### Statistics

All statistical analyses were conducted using Statistical Analysis System (SAS®) release 9.1.3. As no formal hypothesis was tested, intent-to-treat and per-protocol populations were not defined for this study. Laboratory data were summarized using mean, standard deviation (SD), median, minimum, and maximum of the actual values at each assessment. The primary and secondary analyses were conducted using the safety population defined as all patients who entered the study, took at least one dose of the study drug, and had at least one safety follow-up regardless of premature withdrawal.

Patient data for visit 1 week 0 (the baseline) of current study was taken from 33 visit week 104 of core study.

For some data summaries, the baseline values from the core study were used. Analysis in tables and listings are presented overall and by country. AE data were summarized using counts and percentages overall, by intensity, and by relationship to the study drug. Summaries of deaths, withdrawals from study drug, and marked laboratory abnormalities were also presented using counts and percentages.

## Results

### Demographics and baseline disease characteristics

The demographics and baseline disease characteristics were comparable with the pediatric patient population from the core study (CHERISH study). The mean age was 12.0 ± 4.45 years with a range from 4 to 19 years. All patients were white and 80.5% were females (Table 1). Differences in age were reflected in the range of height, weight, and body mass index (BMI). All patients weighed at least 30 kg. The mean duration of pJIA was 4.8 ± 4.08 years. All patients in this study achieved ACR70 at baseline (week 104 of the core study). Demographics and baseline disease characteristics were generally similar in patients from Poland as well as from Russia.

**Table 1:** Demographics and other baseline disease characteristics (safety population)

Characteristics		Poland (*N* = 18)	Russia (*N* = 23)	All Patients (*N* = 41)
Age (years), mean (SD)		12.9 (4.70)	11.3 (4.20)	12.0 (4.45)
Females, *n* (%)		16 (88.9)	17 (73.9)	33 (80.5)
White race, *n* (%)		18 (100)	23 (100)	41 (100)
Height (cm), mean (SD)		149.7 (20.25)	149.6 (22.5)	149.6 (21.29)
Median		158.8	159.0	159.0
Min, max		109.0,174.0	110.0, 184.5	109.0, 184.5
Weight (kg), mean (SD)		44.6 (16.03)	43.3 (19.24)	43.9 (17.70)
Median		49.6	44.0	46.3
Min, max		17.6, 65.0	15.5, 89.7	15.5, 89.7
Body mass index, mean (SD)		19.2 (3.70)	18.3 (3.91)	18.7 (3.80)
Median		19.4	17.4	18.1
Min, max		14.1, 27.6	12.8, 31.4	12.8, 31.4
Disease duration (months), mean (SD)		50.9 (35.53)	62.6 (57.65)	57.5 (48.97)
Joints with active arthritis, mean (SD)		1.3 (2.02)	1.6 (3.15)	1.4 (2.68)
Joints with LOM, mean (SD)		2.8 (5.86)	4.0 (6.71)	3.5 (6.30)
PtGA VAS, mean (SD)		4.7 (7.65)	3.4 (3.65)	4.0 (5.72)
PGA, mean (SD)		5.6 (6.24)	3.7 (3.67)	4.5 (4.99)
CHAQ-DI, mean (SD)		0.14 (0.283)	0.14 (0.209)	0.14 (0.241)
ESR, mean (SD)		6.20 (5.370)	3.90 (2.249)	4.95 (4.084)
CRP, mean (SD)		1.83 (6.107)	0.22 (0.118)	0.95(4.114)

Abbreviations: ACR = American College of Rheumatology; CHAQ-DI = Childhood Health Assessment Questionnaire Disability Index; CRP = C-reactive protein; ESR = erythrocyte sedimentation rate; JIA = juvenile idiopathic arthritis; LOM = limitation of movement; PGA = physician global assessment; PtGA = patient/parent global assessment; SD = standard deviation; VAS = visual analog scale; body mass index = weight (kg) / (height (m))^2^

Demographic data were taken from visit 33 (week 104) of the core study

Concomitant medications were reported for all 18 patients (100%) in Poland and in 16 patients (69.6%) in Russia (Table 2). MTX was the most frequently reported medication (29 patients, 70.7%). In this study, as in the core CHERISH study, the stable doses of MTX (< 20-mg/m^2^ body surface area/week), NSAIDs and low-dose glucocorticoids (no greater than 0.2-mg/kg/day prednisone; daily maximum, 10 mg) were allowed. No other disease modifying drugs were used during the study. The MTX dose reduction depended on the attending physician. The dose might be decreased due to safety reasons or for efficacy in patients who have been in CR for a minimum of 6 months and remained off all corticosteroids.

**Table 2:** Concomitant background medication (MTX, NSAIDs or corticosteroids)

	Poland (*N* = 18)	Russia (*N* = 23)	All patients (*N* = 41)
	*n* (%)	*n* (%)	*n* (%)
Number of patients reporting any concomitant background medication (MTX, NSAIDs or corticosteroids)	18 (100)	15 (65.2)	33 (80.5)
Concomitant MTX	16 (88.9)	13 (56.5)	29 (70.7)
MTX dose summary (mg/week)			
Mean	11.25	10.97	11.15
95% CI	9.42, 13.08	9.26, 12.68	9.87, 12.42
SD	4.541	3.085	4.030
Minimum	2.5	6.0	2.5
Median	10.00	10.00	10.00
Maximum	20.0	15.0	20.0
Concomitant NSAIDs	17 (94.4)	9 (39.1)	26 (63.4)
Concomitant corticosteroids	5 (27.8)	0	5 (12.2)
Corticosteroid dose summary			
Mean	4.25	NC	4.25
95% CI	5.47	NC	3.03
SD	1.591	NC	1.591
Minimum	2.5	NC	2.5
Median	4.00	NC	4.00
Maximum	7.5	NC	7.5

Abbreviations: ATC = anatomical therapeutic chemical classification; CI = confidence interval; MTX = methotrexate; NC = not calculable, NSAIDs = non-steroidal anti-inflammatory drugs; SD = standard deviation; WHO = World Health Organization

WHO drug dictionary version March 2012

Some corticosteroids doses were adjusted to be prednisone equivalent doses

### Safety evaluation

#### Extent of the exposure

The overall mean duration of exposure to TCZ in the study was 55.8 ± 19.40 weeks, together with core study, 159.8 ± 19.40 weeks), and the mean number of infusions was 14.3 ± 4.76 (with core study 47.3 ± 4.76). The total patient duration in the study was 46.41 years (158.41 years with core study).

#### Adverse events

Event rates per 100 patient-years (PY) of exposure were retrospectively calculated for AEs, serious AEs, and infection AEs. Overall, 23 patients (56.1%) experienced 84 events during the study (the event rate of 181.00 AEs per 100 PY). They are presented in Table 3.

**Table 3:** Serious adverse events and adverse events occurring in at least 5% of the patients by treatment group for events

Preferred term	Mild *n* (%) E	Moderate *n* (%) E	Severe *n* (%) E	Life-threatening *n* (%) E	Total *n* (%) E
Patients with any AEs	10 (24.4) 61	12 (29.3) 22	1 (2.4) 1	0	23 (56.1) 84
Peripheral blood	2 (4.9) 5	1 (2.4) 2	0	0	3 (7.3) 7
Neutropenia	2 (4.9) 3	1 (2.4) 1	0	0	3 (7.3) 4
Leukopenia	1 (2.4) 2	1 (2.4) 1	0	0	2 (4.9) 3
Eye disorders	2 (4.9) 2	0	0	0	2 (4.9) 2
Corneal erosion	1 (2.4) 1	0	0		1 (2.4) 1
Corneal opacity	1 (2.4) 1	0	0	0	1 (2.4) 1
Gastrointestinal disorders	2 (4.9) 2	1 (2.4) 1	0	0	3 (7.3) 3
Dyspepsia	1 (2.4) 1	0	0	0	1 (2.4) 1
Periodontitis	0	1 (2.4) 1	0	0	1 (2.4) 1
Vomiting	1 (2.4) 1	0	0	0	1 (2.4) 1
Infections and infestations	8 (19.5) 26	11 (26.8) 15	0	0	19 (46.3) 41
Pharyngitis	4 (9.8) 6	2 (4.9) 2	0	0	6 (14.6) 8
Upper RTI	3 (7.3) 6	2 (4.9) 3	0	0	5 (12.2) 9
Bronchitis	1 (2.4) 1	3 (7.3) 3	0	0	4 (9.8) 4
Acute sinusitis	0	2 (4.9) 3	0	0	2 (4.9) 3
Rhinitis	2 (4.9) 2	0	0	0	2 (4.9) 2
Viral upper RTI	1 (2.4) 1	1 (2.4) 1	0	0	2 (4.9) 2
Blister infected	0	1 (2.4) 1	0	0	1 (2.4) 1
Mononucleosis	0	1 (2.4) 1	0	0	1 (2.4) 1
Oral herpes	1 (2.4) 1	0	0	0	1 (2.4) 1
Otitis media	1 (2.4) 1	0	0	0	1 (2.4) 1
Paronychia	1 (2.4) 1	0	0	0	1 (2.4) 1
Pharyngotonsillitis	0	1 (2.4) 1	0	0	1 (2.4) 1
RTI	1 (2.4) 1	0	0	0	1 (2.4) 1
Tinea versicolour	1 (2.4) 1	0	0	0	1 (2.4) 1
Tonsillitis	1 (2.4) 1	0	0	0	1 (2.4) 1
Urinary infection	1 (2.4) 1	0	0	0	1 (2.4) 1
Varicella	1 (2.4) 1	0	0	0	1 (2.4) 1
Viral infection	1 (2.4) 1	0	0	0	1 (2.4) 1
Viral rhinitis	1 (2.4) 1	0	0	0	1 (2.4) 1
Investigations	2 (4.9) 9	0	1 (2.4) 1	0	3 (7.3) 10
↑ ALT	1 (2.4) 1	0	0	0	1 (2.4) 1
↑ Bilirubin c.	1 (2.4) 2	0	0	0	1 (2.4) 2
↑ Bilirubin	1 (2.4) 2	0	0	0	1 (2.4) 2
↑ CRP	1 (2.4) 1	0	0	0	1 (2.4) 1
↓ Hb	1 (2.4) 1	0	0	0	1 (2.4) 1
↓ Neu	0	0	1 (2.4) 1	0	1 (2.4) 1
↑ ESR	1 (2.4) 1	0	0	0	1 (2.4) 1
↓ WBC	1 (2.4) 1	0	0	0	1 (2.4) 1
Metabolism/nutrition disorders	2 (4.9) 2	0	0	0	2 (4.9) 2
Hypercholesterolemia	1 (2.4) 1	0	0	0	1 (2.4) 1
Hypertriglyceridemia	1 (2.4) 1	0	0	0	1 (2.4) 1
Musculoskeletal/connective tissue disorders	2 (4.9) 2	1 (2.4) 1	0	0	3 (7.3) 3
Back pain	0	1 (2.4) 1	0	0	1 (2.4) 1
Joint swelling	1 (2.4) 1	0	0	0	1 (2.4) 1
Osteoarthritis	1 (2.4) 1	0	0	0	1 (2.4) 1
Nervous system disorders	1 (2.4) 1	1 (2.4) 1	0	0	2 (4.9) 2
Headache	1 (2.4) 1	1 (2.4) 1	0	0	2 (4.9) 2
Renal/urinary disorders	2 (4.9) 6	0	0	0	2 (4.9) 6
Proteinuria	2 (4.9) 3	0	0	0	2 (4.9) 3
Hematuria	1 (2.4) 1	0	0	0	1 (2.4) 1
Leukocyturia	1 (2.4) 2	0	0	0	1 (2.4) 2

Abbreviations: AE = adverse event; E = number of events; MedDRA = medical dictionary for regulatory activities, PT = preferred term, SOC = system organ class, RTI = respiratory tract infection, ALT = alanine aminotransferase, Bilirubin c. = bilirubin conjugated, CRP = C-reactive protein, Hb = hemoglobin, Neu = neutrophil count, ESR = erythrocyte sedimentation rate, WBC = white blood cell count

MedDRA V15.0 coding dictionary

If a patient experienced the same AE preferred term but with different severities, they were counted in the most extreme category only. However, the multiple events of the same AE preferred term were counted in each severity that they occurred. Hence, the total number of events for any one preferred term may exceed the sum of events within individual categories where patient counts exist

##### Adverse events by severity

The majority of patients had AEs that were mild (10 patients, 24.4%) or moderate (12 patients, 29.3%) in intensity, and one patient (2.4%) experienced a severe AE of the decreased neutrophil count (Table 3). Pharyngitis (14.6%) and upper respiratory tract infections (12.2%) were the most frequently AEs occurring. There were three SAEs (7.3%, 6.46 per 100 PY): mild proteinuria, severe neutrophil count decreased, and multiple injuries of moderate severity. There were no deaths, life-threatening AEs (especially malignancies, active TB, or demyelinating disorders) or AEs of special interest during this study. There were a small number of patients with clinically significant abnormal laboratory parameters, the majority of which were isolated cases. One patient had significantly increased liver function tests at the follow-up visit following a reported AE of Epstein-Barr virus infection. Mean values of bilirubin, total cholesterol, low-density lipoprotein cholesterol, high-density lipoprotein cholesterol, and triglyceride concentrations remained stable throughout the study.

##### Adverse events by relationship to the study drug

Overall, there were 12 patients (29.3%) who experienced drug-related AEs that were remotely, probably, or possibly related to TCZ. There were two patients with AEs considered possibly related to TCZ: mild proteinuria and mild decrease in hemoglobin concentration. Both AEs resolved within 5 weeks did not lead to any dose change of the study drug and patients continued the study. The SAE of severe neutrophil count decreased was considered probably related to TCZ; the remaining two SAEs (mild proteinuria and multiple injuries) were considered unrelated.

Nine patients (22.0% of all patients) had an AE that led to dose modifications. There was one patient withdrawn from the treatment due to an AE during the study (severe decrease in neutrophil count). In the remaining eight patients, dose of the drug was omitted due to respiratory tract infections (five patients), dermatitis (two patients), and hyperbilirubinemia (one patient).

### Efficacy evaluation

#### Proportion of patients with JIA ACR 30/50/70/90 responses

Proportions of patients sustaining ACR response at time points to visit 7 (week 24) are presented in the Table 4. All patients in this study achieved ACR70 at baseline (week 104 of the core study). Overall, all patients maintained ACR50 response (using baseline visit of the core study) between baseline (week 104 of the core study) and visit 7 (week 24). Forty patients (97.6%) from 41 treated with TCZ maintained ACR70 response during the study. Only one patient presented the decrease in the level of response from ACR 70 to ACR 50.

**Table 4:** Proportion of patients with JIA ACR30/50/70/90 responses by visit (response calculated based on baseline visit of the core study)

Visit	JIA ACR response	Poland (*N* = 18)	Russia (*N* = 23)	All patients (*N* = 41)
		*n* (%) 95% CI^a^	*n* (%) 95% CI^a^	*n* (%) 95% CI^a^
Visit 1: week 0 (baseline^b^)				
	ACR 30 response	18 (100) 81.5100.0	23 (100) 85.2100.0	41 (100) 91.4, 100.0
	ACR50 response	18 (100) 81.5, 100.0	23 (100) 85.2, 100.0	41 (100) 91.4, 100.0
	ACR70 response	18 (100) 81.5, 100.0	23 (100) 85.2, 100.0	41 (100) 91.4, 100.0
	ACR90 response	11 (61.1) 35.7, 82.7	19 (82.6) 61.2, 95.0	30 (73.2) 57.1, 85.8
Visit 4: week 12				
	ACR 30 response	18 (100) 81.5, 100.0	23 (100) 85.2, 100.0	41 (100) 91.4, 100.0
	ACR50 response	18 (100) 81.5, 100.0	23 (100) 85.2, 100.0	41 (100) 91.4, 100.0
	ACR70 response	18 (100) 81.5, 100.0	23 (100) 85.2, 100.0	41 (100) 91.4, 100.0
	ACR90 response	12 (66.7) 41.0, 86.7	20 (87.0) 66.4, 97.2	32 (78.0) 62.4, 89.4
Visit 7: week 24				
	ACR 30 response	18 (100) 81.5, 100.0	23 (100) 85.2, 100.0	41 (100) 91.4, 100.0
	ACR50 response	18 (100) 81.5, 100.0	23 (100) 85.2, 100.0	41 (100) 91.4, 100.0
	ACR70 response	17 (94.4) 72.7, 99.9	23 (100) 85.2, 100.0	40 (97.6) 87.1, 99.9
	ACR90 response	13 (72.2) 46.5, 90.3	20 (87.0) 66.4, 97.2	33 (80.5) 65.1, 91.2

Abbreviations: ACR = American College of Rheumatology; CI = confidence interval; JIA = juvenile idiopathic arthritis

^a^Exact 2-sided CI (Clopper and Pearson) based upon the observed proportion of patients

^b^Baseline is the baseline visit of study and uses visit 33 (week 104) of the core study. For some patients, re-screening value was used as baseline to consider the last available non-missing value prior to the start of treatment

#### Proportion of patients with inactive disease at visits

The proportion of patients with inactive disease at selected visits is provided in Table 5. Overall, the number of patients who achieved inactive disease increased between baseline and visit 7 (week 24) from 26 patients (63.4%) to 31 (75.6%), respectively.

**Table 5:** Proportion of patients with inactive disease by visit (safety population)

Visit	Poland (*N* = 18)	Russia (*N* = 23)	All patients (*N* = 41)
	*n* (%) 95% CI^a^	*n* (%) 95% CI^a^	*n* (%) 95%
Visit 1: week 0 (baseline)			
Inactive disease	11 (61.1) 35.7, 82.7	15 (65.2) 42.7, 83.6	26 (63.4) 46.9, 77.9
Signs of still active disease	7 (38.9) 17.3, 64.3	8 (34.8) 16.4, 57.3	15 (36.6) 22.1, 53.1
Visit 4: week 12			
Inactive disease	11 (61.1) 35.7, 82.7	16 (69.6) 47.1, 86.8	27 (65.9) 49.4, 79.9
Signs of still active disease	7 (38.9) 17.3, 64.3	7 (30.4) 13.2, 52.9	14 (34.1) 20.1, 50.6
Visit 7: week 24			
Inactive disease	11 (61.1) 35.7, 82.7	20 (87.0) 66.4, 97.2	31 (75.6) 59.7, 87.6
Signs of still active disease	7 (38.9) 17.3, 64.3	3 (13.0) 2.8, 33.6	10 (24.4) 12.4, 40.3

Abbreviations: CI = confidence interval;

Week 0 data were taken from visit 33 (week 104) of the core study

^a^Exact 2-sided CI (Clopper and Pearson) based upon the observed proportion of patients

#### Proportion of patients with clinical remission at visits

The proportion of patients with CR at time points to visit 7 (week 24) is provided in Table 6. Overall, the number of patients who achieved CR increased between baseline and visit 7 (week 24) from 18 (43.9%) patients to 20 (48.8%), respectively. The most frequent level of the response observed was level 1 and level 2.

**Table 6:** Proportion of patients with clinical remission by visit (safety population)

Visit	Poland (*N* = 18)	Russia (*N* = 23)	All patients (*N* = 41)
	*n* (%) 95% CI^a^	*n* (%) 95% CI^a^	*n* (%) 95% CI^a^
Visit 1: week 0 (baseline)			
Clinical remission	8 (44.4) 21.5, 69.2	10 (43.5) 23.2, 65.5	18 (43.9) 28.5, 60.3
Level 1	1 (5.6) 0.1, 27.3	2 (8.7) 1.1, 28.0	3 (7.3) 1.5, 19.9
Level 2	7 (38.9) 17.3, 64.3	6 (26.1) 10.2, 48.4	13 (31.7) 18.1, 48.1
Level 3	0 0.0, 18.5	1 (4.3) 0.1, 21.9	1 (2.4) 0.1, 12.9
Level 4	0 0.0, 18.5	1 (4.3) 0.1, 21.9	1 (2.4) 0.1, 12.9
Visit 4: week 12			
Clinical remission	9 (50.0) 26.0, 74.0 1	0 (43.5) 23.2, 65.5	19 (46.3) 30.7, 62.6
Level 1	2 (11.1) 1.4, 34.7	2 (8.7) 1.1, 28.0	4 (9.8) 2.7, 23.1
Level 2	7 (38.9) 17.3, 64.3	6 (26.1) 10.2, 48.4	13 (31.7) 18.1, 48.1
Level 3	0 0.0, 18.5	1 (4.3) 0.1, 21.9	1 (2.4) 0.1, 12.9
Level 4	0 0.0, 18.5	1 (4.3) 0.1, 21.9	1 (2.4) 0.1, 12.9
Visit 7: week 24			
Clinical remission	10 (55.6) 30.8, 78.5	10 (43.5) 23.2, 65.5	20 (48.8) 32.9, 64.9
Level 1	3 (16.7) 3.6, 41.4	2 (8.7) 1.1, 28.0	5 (12.2) 4.1, 26.2
Level 2	7 (38.9) 17.3, 64.3	6 (26.1) 10.2, 48.4	13 (31.7) 18.1, 48.1
Level 3	0 0.0, 18.5	1 (4.3) 0.1, 21.9	1 (2.4) 0.1, 12.9
Level	0 0.0, 18.5	1 (4.3) 0.1, 21.9	1 (2.4) 0.1, 12.9

Abbreviations: CI = confidence interval; level 1 = clinical remission on medication; level 2 = clinical remission off oral corticosteroid medication (still on TCZ); level 3 = clinical remission off both oral corticosteroid and MTX medication (still on TCZ); level 4 = clinical remission off all anti-inflammatory medications (still on TCZ); week 0 data were taken from visit 33 (week 104) of the core study

^a^Exact 2-sided CI (Clopper and Pearson) based upon the observed proportion of patients

## Discussion

Results of this first observational, long-term study on TCZ in patients with pJIA demonstrate that continuing treatment over 104 to 131 weeks or longer with intravenous TCZ (8 mg/kg administered every 4 weeks) is safe and efficacious for the management of pJIA. The safety and efficacy of TCZ for the treatment of pJIA were demonstrated in several only in few studies, although their follow-up periods were shorter than in our study [5–7].

The overall incidence of AE and SAEs rates during the follow-up period of this study was found to be lower to the results of the core study, 181.0 and 6.46, respectively. The event rate of infection AEs was 88.34/100 PY. Pharyngitis and upper respiratory tract infection were the most common AE: 14.6 and 12.2%, respectively, in the core study 10.1 and 9.0%, respectively. The frequency of neutropenia was 7.3%, in core study 3.7%. Hypercholesterolemia occurred in one child, whereas in the core study in 34.6%. With the exception of one patient who had severe neutropenia that led to withdrawal from the study, all other AEs were either mild (24.4%) or moderate (29.3%). There were no deaths, life-threatening AEs, or AEs of specific side effect. The majority of AEs were unrelated to the study drug.

These results are in accordance with Imagawa et al. open-labeled 48-week study of TCZ treatment in 19 pJIA patients [6]. The most common AEs were nasopharyngitis (nine events, 47.4% of patients) and upper respiratory tract infection (nine events, 47.4%). Four patients required hospitalization due to SAEs: gastroenteritis (*n* = 2), pneumonia (*n* = 1), and sensory disturbance (*n* = 1). All laboratory abnormalities were mild in severity [6].

Horneff et al. [7] observed during 72.4 PY rare serious or medically important infections in the TCZ cohort included one case each of pneumonia, appendicitis, and influenza. No cases of TB occurred, and apart from herpes zoster, no opportunistic infections were observed. Only two patients discontinued TCZ due to intolerance. These results indicate a good tolerance of treatment that does not change during long-term administration. The awareness of AEs and treatment experience results in increased attention in observation of the patient.

As was already mentioned, the secondary objective of the study was to investigate the long-term efficacy of treatment with TCZ in patients with pJIA. All patients in this study achieved ACR70 at baseline (week 104 of the core study) and continued to achieve at least ACR50 (one patient) or ACR70 (40 patients) response up to week 24 of this long-term extension study using baseline visit of the core study. Other secondary efficacy parameters showed similar patterns of the continued response across the study. During the CHERISH core study, results were clinically meaningful because a high proportion (89%) of patients achieved JIA-ACR30 response by week 16; 62% of patients achieved JIA-ACR70 response, and 26% even achieved JIA-ACR90 response. In the double-blind, withdrawal phase, JIA flares occurred in 48.1% of patients on placebo versus in 25.6% receiving TCZ. At the end of part 2 of the study, 64.6 and 45.1% of patients receiving TCZ obtained JIA ACR 70 and JIA ACR 90 responses, respectively, which confirmed the high efficacy of the treatment. In Imagawa et al. report, the ACR Pedi 70 response rate reached 94.1% (16/17) at week 24 and remained high through week 48 [6].

In Horneff et al. observation [7] in 74 patients with mean treatment duration 0.98 ± 0.59 year in TCZ cohort, the improvement according to Ped ACR30/50/70/90 criteria was reached after 3 months by 61%/52%/35%/26% patients. At 24 months, JADAS minimal disease activity was achieved in 52.4% and JADAS remission in 27.9% patients [7]. These reports also indicate the effectiveness of the therapy, but the comparison is limited due to the different duration of treatment and the method of assessment.

Presented study allowed for the acquisition of efficacy and safety data during treatment with TCZ for 131 or more weeks in children with pJIA. The potential limitation of the study is the different duration of TCZ therapy, although efficacy was assessed after the same treatment period.

The question is how long patients should receive biological agent. It is unclear whether patients with JIA who achieved remission should to continue biological treatment. Our study evaluated the efficacy of TCZ in children who did not respond to monotherapy with MTX. Guzman et al. [12] reported that the probability of attaining remission off medication in JIA within 5 years during the first 5 years of the disease was 46–57% across JIA categories except for polyarthritis: for RF-positive—0% and for RF-negative—14% [12]. Moreover, based on the study by Ghiti et al., performed on adult rheumatoid arthritis (RA) patients, stopping an anti-TNF treatment was associated with substantially more flares of the disease than in RA patients with remission or with stable low disease activity continuing this biological therapy [13].

Our study demonstrated that longer duration of drug administration did not affect the incidence of AEs. Also, maintained response to the study drug was observed. The efficacy response and the consistent safety profile support the use of TCZ in pJIA. This is particularly important for new therapies in children.

## Conclusions

This is the first, observational, long-term study with TCZ in pJIA in children. Our data demonstrate that continuing treatment over 104 to 131 weeks or longer with TCZ is safe and efficacious for the management of pJIA. The safety profile of TCZ in this population was consistent with that seen in the core study. The majority of AEs were unrelated to TCZ and were consistent with the pediatric and pJIA population. The presented data confirm the long-term safety of TCZ use in patients with pJIA. The efficacy response remained stable to 131 week of treatment or longer of this long-term extension study. Taken together, the continued efficacy response and the consistent safety profile support the use of TCZ in pJIA.

## References

[1] Petty RE, Southwood TR, Manners P, Baum J, Glass DN, Goldenberg J, He X (2004). International League of Associations for Rheumatology classification of juvenile idiopathic arthritis: second revision, Edmonton, 2001. J Rheumatol.

[2] Ringold S, Seidel KD, Koepsell TD, Wallace CA (2009). Inactive disease in polyarticular juvenile idiopathic arthritis: current patterns and associations. Rheumatology (Oxford).

[3] Wallace CA, Huang B, Bandeira M, Ravelli A, Giannini EH (2005). Patterns of clinical remission in select categories of juvenile idiopathic arthritis. Arthritis Rheum.

[4] Webb K, Wedderburn LR (2015). Advances in the treatment of polyarticular juvenile idiopathic arthritis. Curr Opin Rheumatol.

[5] Brunner HI, Ruperto N, Zuber Z, Keane C, Harari O, Kenwright A (2015). Efficacy and safety of tocilizumab in patients with polyarticular-course juvenile idiopathic arthritis: results from a phase 3, randomised, double-blind withdrawal trial. Ann Rheum Dis.

[6] Imagawa T, Yokota S, Mori M, Miyamae T, Takei S, Imanaka H (2012). Safety and efficacy of tocilizumab, an anti-IL-6-receptor monoclonal antibody, in patients with polyarticular-course juvenile idiopathic arthritis. Mod Rheumatol.

[7] Horneff G, Klein A, Klotsche J (2016). Comparison of treatment response, remission rate and drug adherence in polyarticular juvenile idiopathic arthritis patients treated with etanercept, adalimumab or tocilizumab. Arthritis Res Ther.

[8] De Benedetti F, Brunner HI, Ruperto N, Kenwright A, Wright S, Calvo I (2012). Randomized trial of tocilizumab in systemic juvenile idiopathic arthritis. N Engl J Med.

[9] Turnier JL, Brunner HI (2016). Tocilizumab for treating juvenile idiopathic arthritis. Expert Opin Biol Ther.

[10] Giannini EH, Ruperto N, Ravelli A, Lovell DJ, Felson DT, Martini A (1997). Preliminary definition of improvement in juvenile arthritis. Arthritis Rheum.

[11] Wallace CA, Ruperto N, Giannini E (2004). Preliminary criteria for clinical remission for select categories of juvenile idiopathic arthritis. J Rheumatol.

[12] Guzman J, Oen K, Tucker LB (2015). The outcomes of juvenile idiopathic arthritis in children managed with contemporary treatments: results from the ReACCh-Out cohort. Ann Rheum Dis.

[13] Ghiti Moghadam M, Vonkeman HE, Ten Klooster PM, Dutch National POET Collaboration (2016). Stopping tumor necrosis factor inhibitor treatment in patients with established rheumatoid arthritis in remission or with stable low disease activity: a pragmatic multicenter, open-label randomized controlled trial. Arthritis Rheumatol.

